# Clinicopathological Prognostic Factors After Pulmonary Metastasectomy for Bone and Soft-Tissue Sarcoma: A Single-Centre Retrospective–Prospective Cohort Study

**DOI:** 10.3390/cancers18183054

**Published:** 2026-09-20

**Authors:** Elisabet Martínez Téllez, José Belda Sanchís, Sonia Stephania Chavarría Murillo, Georgina Ros Brasó, Alejandra Libreros Niño, Jorge Hernández Ferrández, Georgina Planas Cánovas, Marina Paradela de la Morena, Juan Carlos Trujillo Reyes

**Affiliations:** 1 Thoracic Surgery Department, Hospital de la Santa Creu i Sant Pau, 08041 Barcelona, Spain; jbeldas@santpau.cat (J.B.S.); schavarria@santpau.cat (S.S.C.M.); gros@santpau.cat (G.R.B.); elibreros@santpau.cat (A.L.N.); jhernandezf@santpau.cat (J.H.F.); gplanas@santpau.cat (G.P.C.); paradeladelamorena@gmail.com (M.P.d.l.M.); jtrujillo@santpau.cat (J.C.T.R.); 2 Thoracic Surgery Research and Innovation in Lung Cancer (TRAIL Research Group), Institut de Recerca Sant Pau, 08041 Barcelona, Spain

**Keywords:** sarcoma, pulmonary metastasectomy, prognostic factors, overall survival, disease-free survival, R0 resection, disease-free interval, lymphadenectomy, oligometastatic disease, thoracic surgery

## Abstract

**Simple Summary:**

Sarcomas are rare cancers that often spread to the lungs. Surgery to remove the lung metastases is frequently offered, but it is still unclear which patients truly benefit and which are exposed to an operation that will not change the course of their disease. We reviewed 57 patients who underwent 80 lung operations for sarcoma metastases at a European reference centre, looking for features linked to how long patients lived and how long they stayed free of disease. Two mattered most: whether the surgeon was able to remove all the tumour, and how soon the metastases had appeared after the original cancer was diagnosed. Involvement of the lymph nodes inside the chest, undetectable before surgery, was also linked to shorter survival. The type of sarcoma, the surgical approach and the number of metastases removed made no difference. These findings describe how patients already selected for surgery fared, and may help refine expectations and risk stratification once metastasectomy is being considered, rather than establish who would, or would not, benefit from surgery as opposed to non-surgical management.

**Abstract:**

**Background/Objectives:** Pulmonary metastasectomy is widely performed in sarcoma, yet patient selection remains empirical and no randomised evidence supports it. We aimed to identify clinicopathological factors associated with overall survival (OS) and disease-free survival (DFS) after pulmonary metastasectomy for bone and soft-tissue sarcoma. **Methods:** Observational single-centre cohort study combining a retrospective phase (June 2014–December 2016) and a prospective phase (January 2017–January 2021), with follow-up closed in January 2022. Survival was estimated using the Kaplan–Meier method and compared by log-rank testing; variables with *p* < 0.2 entered a multivariable Cox model selected by the Akaike information criterion. **Results:** Fifty-seven patients (34 men; mean age 52.8 ± 14.0 years) underwent 80 metastasectomies; 46 (80.7%) had soft-tissue and 11 (19.3%) bone sarcoma. Complete (R0) resection was achieved in 73 procedures (91.2%). Morbidity was 13.8%, with no 30-day readmission or mortality. After a median follow-up of 54.0 months, median OS was 34.0 months (1-, 3- and 5-year OS 82.5%, 46.7% and 32.6%) and median DFS 7.0 months. Incomplete resection was the factor most strongly associated with adverse outcome for both OS (HR 6.54, 95% CI 2.49–17.14) and DFS (HR 8.66, 3.26–23.03). Early metastatic appearance was independently associated with worse OS (HR 2.60, 1.20–5.65) and synchronous presentation with earlier recurrence (HR 2.54, 1.24–5.19). Occult nodal involvement, present in 6.5% of assessed procedures, was associated with survival univariably (*p* = 0.010). Histological subtype, surgical approach, extent of resection and number of nodules resected had no independent effect. **Conclusions:** Completeness of resection is the factor most strongly associated with outcome. Early metastatic appearance identifies patients in whom surgery alone is unlikely to be sufficient, and nodal staging should accompany metastasectomy to better estimate the prognosis.

## 1. Introduction

Up to half of patients with a sarcoma of bone or soft tissue will develop distant metastases, and the lung is by far the dominant target organ. Haematogenous dissemination through the pulmonary capillary bed makes isolated lung involvement the most common pattern of failure, and in a substantial proportion of patients the lung remains the only site of disease for a prolonged period.

Surgical resection of pulmonary metastases has become the second most frequent indication for lung resection in thoracic surgery departments, after resection for primary bronchogenic carcinoma [1]. Its diffusion has, however, outpaced the evidence supporting it. The International Registry of Lung Metastases established in 1997 the three variables still used today—completeness of resection, disease-free interval and number of metastases—but did so from a heterogeneous, uncontrolled registry [2]. Two decades later, the literature comprises more than 500 papers, almost all retrospective series of fewer than 100 highly selected patients accrued over long periods, with heterogeneous primary tumours and no comparator arm [3]. A systematic review of reported outcomes in sarcoma, benchmarked against population-based registry data, concluded that the apparent survival advantage of metastasectomy is at least partly attributable to selection rather than to the operation itself [4]. No randomised trial has been completed in sarcoma.

This leaves the clinician with a concrete problem. Current European guidelines for soft-tissue and for bone sarcoma acknowledge surgery of lung metastases as an option in selected patients but do not define the selection criteria operationally [5,6]. Surgery is therefore offered on the basis of criteria—controlled primary tumour, resectable disease, adequate physiological reserve—that define feasibility rather than benefit. Which patients derive an actual survival advantage, and which undergo a metastasectomy that does not alter the natural history of their disease, remains poorly defined. Several specific questions remain unsettled: whether histological subtype should influence the decision; whether occult hilar or mediastinal nodal involvement carries the same adverse prognostic weight as in primary lung cancer, and therefore whether systematic lymphadenectomy is warranted either to better estimate the prognosis or as part of the treatment; whether a minimally invasive approach compromises oncological completeness given the well-documented radiological–pathological discrepancy in sarcoma; and where the threshold for disease-free interval should be placed.

We therefore analysed a consecutive cohort of patients undergoing pulmonary metastasectomy for sarcoma at a Spanish national reference centre (CSUR) and member of the European Reference Network for rare adult solid cancers (ERN-EURACAN). A series accrued within a reference network offers two things the wider literature largely lacks: a single, stable multidisciplinary indication policy applied unchanged across the whole study period, and systematic pathological assessment of hilar and mediastinal nodes. Our aim was to identify clinicopathological factors associated with overall survival (OS) and disease-free survival (DFS), and to examine separately the prognostic contribution of histological subtype, nodal involvement, surgical approach, extent of resection, metastatic synchronicity and repeat metastasectomy.

## 2. Materials and Methods

### 2.1. Study Design and Setting

This was an observational, descriptive, single-centre cohort study with a sequential retrospective and prospective design. Patients were assessed by the multidisciplinary team of the Functional Unit of Mesenchymal Tumours of Hospital de la Santa Creu i Sant Pau (Barcelona, Spain)—a Spanish national reference centre (CSUR) and an ERN-EURACAN member—and operated on in the Department of Thoracic Surgery of the same institution.

The retrospective phase covered June 2014 to December 2016 and the prospective phase January 2017 to January 2021, the study protocol having been submitted for ethical review before the latter began. Follow-up was closed in January 2022, so that the last patient included had a minimum of twelve months of follow-up. No formal sample size calculation was performed; all consecutive patients meeting the eligibility criteria during the study period were included.

The study was reported in accordance with the Strengthening the Reporting of Observational Studies in Epidemiology (STROBE) guidelines (Table S1).

### 2.2. Eligibility Criteria

Patients aged 12–90 years with a histologically confirmed extrapulmonary sarcoma and one or more pulmonary nodules diagnosed as, or highly suspicious for, sarcoma metastases were eligible, provided the primary tumour was locally controlled and surgery was expected a priori to achieve complete resection of all detectable disease. Patients with an uncontrolled primary tumour, with extrapulmonary metastatic recurrence not amenable to surgical treatment, or with comorbidity precluding surgery were excluded.

For the retrospective phase, all patients operated on for pulmonary metastases with a diagnosis of extrapulmonary sarcoma were pre-selected from the departmental database; the clinical record and the pathology report of the resected lung specimens were then reviewed in every case to confirm both the sarcomatous nature of the primary tumour and of the pulmonary lesions. Patients operated on for benign lesions, primary lung tumours or metastases of non-sarcomatous origin were excluded on this basis. For the prospective phase, consecutive patients fulfilling the criteria were enrolled after multidisciplinary discussion.

### 2.3. Criteria for Pulmonary Metastasectomy

Every candidate was discussed by the multidisciplinary team (MDT) of the Functional Unit of Mesenchymal Tumours, which issued the final recommendation for surgery. The criteria applied were: (i) histologically confirmed sarcoma with the primary tumour locally controlled at the time of the thoracic indication; (ii) disease confined to the lung on preoperative staging, extrapulmonary metastatic disease being excluded unless itself amenable to radical treatment; (iii) all pulmonary lesions judged technically resectable with an expected R0 result and preservation of sufficient functioning parenchyma; (iv) cardiopulmonary reserve sufficient to tolerate the planned resection, assessed by spirometry, DLCO and, where indicated, cardiological evaluation; and (v) absence of comorbidity precluding general anaesthesia and thoracic surgery.

Staging comprised thin-section thoracic CT in all cases, with FDG-PET/CT used according to histology and to the protocol of the primary site, principally to exclude extrapulmonary disease. Mediastinal nodal staging was not performed routinely: EBUS or PET/CT was requested only where imaging raised the suspicion of nodal disease, and no patient in this cohort was operated on with radiologically suspected nodal involvement.

Neither the number of pulmonary metastases nor a bilateral distribution was in itself a formal contraindication, provided complete resection of all detectable disease was considered achievable, if necessary through staged procedures. Previous systemic therapy or radiotherapy, whether for the primary tumour or for the metastatic disease, was not a contraindication to surgery. The same criteria were applied to candidates for repeat metastasectomy. Importantly, neither the indication criteria nor the staging work-up changed between the retrospective and the prospective phases of the study.

No procedure was excluded from the analysis on the basis of its pathological result: the seven operations that ended in an R1 or R2 resection were retained and analysed according to the intention at the time of the indication. No unexpected pleural metastatic deposits were encountered intraoperatively in any procedure of this series.

### 2.4. Variables and Definitions

Variables were collected in five domains: patient characteristics; primary tumour and its treatment; characteristics of the pulmonary metastases; the metastasectomy procedure; and follow-up.

Metastases were classified as synchronous when diagnosed at the same time as the primary tumour and metachronous when diagnosed subsequently. The disease-free interval (DFI) was defined as the time in months from diagnosis of the primary tumour to diagnosis of the first pulmonary metastasis, and was dichotomised at 12 months on the basis of previous reports in which this threshold discriminated survival [7,8,9]. The number of resected nodules was categorised as 1, 2–3, 4–10 and >10, and dichotomised at three for survival analysis; the largest resected nodule was dichotomised at 3 cm [9]. Preoperative FEV1 and DLCO were dichotomised at 80% of predicted.

Completeness of resection was classified as R0 (no macroscopic or microscopic residual disease), R1 (microscopic residual disease) or R2 (macroscopic residual disease), and margin status was recorded separately from the definitive pathology report. Nodal assessment was categorised as complete hilar–mediastinal lymphadenectomy, nodal sampling, or no nodal biopsy, and the involved station recorded as hilar, mediastinal, or hilar and mediastinal. For survival analyses, in which the unit of observation is the patient, nodal involvement was defined from the status recorded at the index (first) metastasectomy; findings at repeat procedures were not used to reclassify a patient’s status. Complications were graded according to the Clavien–Dindo classification [10], with grades I–II considered minor and III–IV major.

Overall survival (OS) was measured from the date of metastasectomy to death from any cause or last follow-up. Disease-free survival (DFS) was measured from the date of metastasectomy to the first recurrence, pulmonary or extrapulmonary, whichever occurred first. The date of metastasectomy was the date of entry into the study.

Follow-up after metastasectomy consisted of clinical assessment and thoracic CT every three months during the first two years, every six months until the fifth year, and annually thereafter. The same schedule was applied throughout the study period.

### 2.5. Data Sources and Management

Data were extracted from the institutional electronic record (SAP Patients: SAP ERP 6.0 EHP 6 + IS-H 606, in use since 2011), which holds discharge summaries, operative notes, radiology and pathology reports. Vital status at study closure was verified in the institutional record or, failing that, in the CatSalut Historial Electrònic de Salut; where neither was informative, the patient was contacted by telephone. Data were entered into a purpose-designed spreadsheet with a unique identifier per patient and imported into IBM SPSS Statistics v22.0 (IBM Corp., Armonk, NY, USA) for analysis. No patient was lost to oncological follow-up.

### 2.6. Statistical Analysis

Quantitative variables are summarised as mean, standard deviation, 95% confidence interval of the mean, median, range and interquartile range; qualitative variables as absolute and relative frequencies. Normality was assessed with the Kolmogorov–Smirnov test. Homogeneity between the bone and soft-tissue sarcoma cohorts, and between synchronous and metachronous presentations, was tested with Fisher’s exact test for binary variables, the chi-square test for variables with more than two categories, Student’s *t*-test or the Mann–Whitney U test for continuous variables according to distribution, and Spearman’s correlation for ordinal variables.

The primary endpoint was the identification of prognostic factors for OS; the secondary endpoint, prognostic factors for DFS. For both, survival curves were generated by the Kaplan–Meier method for the following candidate factors: sarcoma type (bone vs. soft tissue), timing of metastases (metachronous vs. synchronous), DFI (<12 vs. ≥12 months), number of pulmonary nodules (1–3 vs. >3), surgical approach (thoracotomy vs. VATS), completeness of resection (R0 vs. R1–R2), margin status, nodal involvement, previous non-sarcomatous malignancy, primary tumour site (extremity vs. non-extremity), neoadjuvant and adjuvant chemotherapy, neoadjuvant and adjuvant radiotherapy, and laterality. Curves were compared by the log-rank test.

Variables with *p* < 0.2 on univariable analysis were entered into a multivariable Cox proportional hazards model, and the final model was selected on the basis of the lowest Akaike information criterion. This strategy is intended to retain variables carrying prognostic information rather than to test hypotheses, and consequently some retained variables have *p* > 0.05; these are reported as retained in the model and are not described as prognostic factors. Hazard ratios are reported with 95% confidence intervals, and only associations whose confidence interval excludes unity are interpreted as demonstrating an independent effect. Statistical significance was set at *p* < 0.05.

Two decisions were taken a priori in the specification of the multivariable models. First, nodal involvement was not entered into the models. Although strongly associated with survival on univariable analysis, it was observed in only four patients, and estimating a hazard ratio from four events produces sparse-data bias, an upwardly biased coefficient with an uninterpretably wide confidence interval; nodal involvement is therefore reported descriptively and univariably only. Second, synchronicity and dichotomised DFI were never entered as separate covariates in the same model, because synchronous disease has by definition a disease-free interval of zero and the two variables are therefore structurally collinear. Timing of metastatic appearance was instead represented by a single variable in each model, the parameterisation being chosen by the same Akaike criterion applied to the rest of the model: early metastatic appearance (synchronous disease or a DFI < 12 months, versus a DFI ≥ 12 months) for OS, and synchronous versus metachronous presentation for DFS.

Two patients whose recurrence was recorded in the same month as surgery were assigned a disease-free survival time of half a month, so that they could be retained in all analyses. Proportional hazards assumptions were assessed by inspection of the log-minus-log survival plots and by testing the scaled Schoenfeld residuals. Models were restricted so that at least ten events were available per variable estimated. Applying the Akaike criterion to the candidate variables retained two covariates in each final model, against 31 deaths for OS and 48 recurrence events for DFS. The number of nodules resected was screened into the OS model but excluded by the Akaike criterion, and adding a separate disease-free interval term to the DFS model, alongside synchronicity, likewise worsened model fit; both are therefore reported univariably only. Disease-free survival is reported to three years, the maximum duration for which recurrence status was systematically ascertained in this cohort; five-year estimates are not presented. Neoadjuvant and adjuvant chemotherapy and radiotherapy, and primary tumour site, were subsequently added to this candidate list (Section 3.6 and Section 3.7); because two chemotherapy variables met the *p* < 0.2 screening threshold, we additionally report, as a post hoc sensitivity analysis reported separately from the pre-specified models of Table 1 and Table 2, their effect when added to the final models (Section 3.8).

The multivariable models were deliberately restricted to a small number of covariates because of the limited number of events; the objective was a parsimonious and interpretable model rather than exhaustive adjustment.

### 2.7. Ethics

This study was conducted in accordance with the Declaration of Helsinki. The protocol was submitted to the Ethics Committee of Hospital de la Santa Creu i Sant Pau before the start of prospective enrolment and was approved on 7 April 2017 (protocol code IIBSP-SAR-2016-97). Written informed consent was obtained from all participants, or from their legal representative where the patient was unable to give it. No biological samples were collected. Data confidentiality was handled in accordance with the applicable Spanish data-protection legislation.

## 3. Results

### 3.1. Patient and Primary Tumour Characteristics

Between June 2014 and January 2021, 57 patients underwent 80 pulmonary metastasectomy procedures, 26 in the retrospective and 54 in the prospective period. There were 34 men (59.6%) and 23 women (40.4%), with a mean age at first metastasectomy of 52.8 ± 14.0 years; 3% had a history of a previous unrelated malignancy.

Forty-six patients (80.7%) had a soft-tissue sarcoma and 11 (19.3%) a bone sarcoma; leiomyosarcoma was the commonest soft-tissue subtype (n = 16, 35%) and conventional osteosarcoma the commonest bone subtype (n = 5). The primary tumour arose in the extremities in 63.6% of bone and 52.2% of soft-tissue sarcomas, and mean primary tumour size was 10.3 ± 6.3 cm (data available for 54 patients). Patient and primary tumour characteristics, including neoadjuvant and adjuvant chemotherapy and radiotherapy, are summarised in Table 3.

### 3.2. Characteristics of the Pulmonary Metastases

Metastases were metachronous in 46 patients (80.7%) and synchronous in 11 (19.3%); among metachronous cases, the median disease-free interval was 23 months, and 11 patients (23.9%) had a DFI below 12 months.

Disease was bilateral in 15 patients (26.3%), of whom 9 (60%) underwent staged bilateral metastasectomy; in the remaining 6, the contralateral operation was abandoned because of radiological progression between procedures. Preoperative FEV1 was at least 80% of predicted in 85.5% of patients and DLCO at least 80% in 70%. The number and size of the resected nodules are given in Table 4.

### 3.3. Surgical Procedures

Fifty of the eighty procedures (62.5%) were performed through thoracotomy and twenty (25%) by VATS, with five rethoracotomies and five redo-VATS; no robotic procedure was performed. The distribution shifted markedly over time: none of the 26 procedures performed in 2014–2016 was minimally invasive, against 46.3% of the 54 performed in 2017–2021.

Most resections were non-anatomical wedge resections; ten lobectomies, three bilobectomies and three en bloc resections of the diaphragm and/or pericardium were also performed, and no pneumonectomy. Resection was complete (R0) in 73 procedures (91.2%), and margins were negative in 91.2% of definitive pathology reports.

Nodal tissue was assessed in 61 procedures, by complete hilar–mediastinal lymphadenectomy in 59 and by sampling in 2; of the 19 procedures without nodal biopsy, 9 followed a lymphadenectomy performed at a previous operation. Nodal involvement, not suspected on preoperative imaging, was found in 4 of the 61 assessed procedures (6.5%): hilar only in 2 patients and hilar plus mediastinal in 2. The corresponding primary tumours were two synovial sarcomas, one myxofibrosarcoma and one malignant fibrous histiocytoma. Surgical variables are summarised in Table 4.

### 3.4. Perioperative Outcomes

Mean hospital stay was 4.2 ± 3.1 days (median 4 days, 95% CI 3.5–4.9) and chest drains were removed at a median of 2.5 days. Complications followed 11 of 80 procedures (13.8%), most often prolonged air leak beyond the fifth postoperative day (n = 5); by Clavien–Dindo grade there were six grade I, one grade II and four grade III events and no grade IV, with no readmission and no death within 30 days of surgery.

### 3.5. Recurrence

Recurrence at any site occurred in 48 of the 57 patients (84.2%) during follow-up, at a mean of 8.4 ± 9.8 months and a median of 6 months (95% CI 5.7–11.1). Pulmonary recurrence occurred in 38 patients at a median of 6 months (95% CI 5.6–12.0), of whom 14 underwent a further operation; the median interval between consecutive operations was 11 months (95% CI 9.6–14.5).

### 3.6. Overall Survival: Univariable Analysis

**Figure 1:**
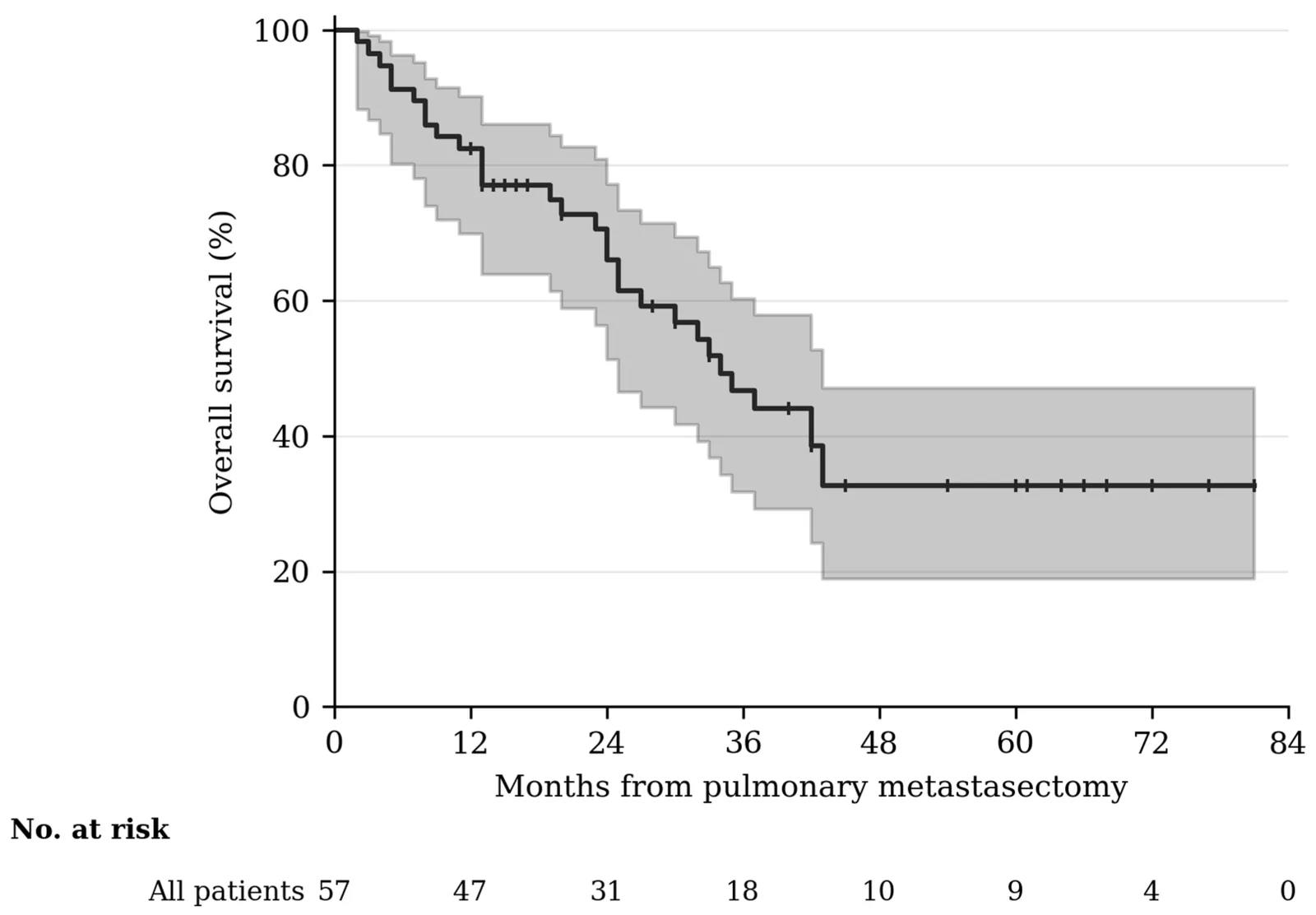
Kaplan–Meier estimate of overall survival for the whole cohort after pulmonary metastasectomy (with shadows lines reflecting 95% CI). Vertical marks denote censored observations.

At the census date, 31 of the 57 patients (54.4%) had died. Median follow-up estimated by the reverse Kaplan–Meier method was 54.0 months. For the whole cohort, median OS after metastasectomy was 34.0 months, with 1-, 3- and 5-year OS of 82.5%, 46.7% and 32.6%, respectively (Figure 1).

Median OS was 43.0 months for bone sarcoma and 33.0 months for soft-tissue sarcoma, a non-significant difference (*p* = 0.50). Synchronicity considered on its own had no effect (32.0 vs. 35.0 months for synchronous vs. metachronous; *p* = 0.92), nor did surgical approach (35.0 months for thoracotomy vs. 33.0 months for VATS; *p* = 0.80).

**Figure 2:**
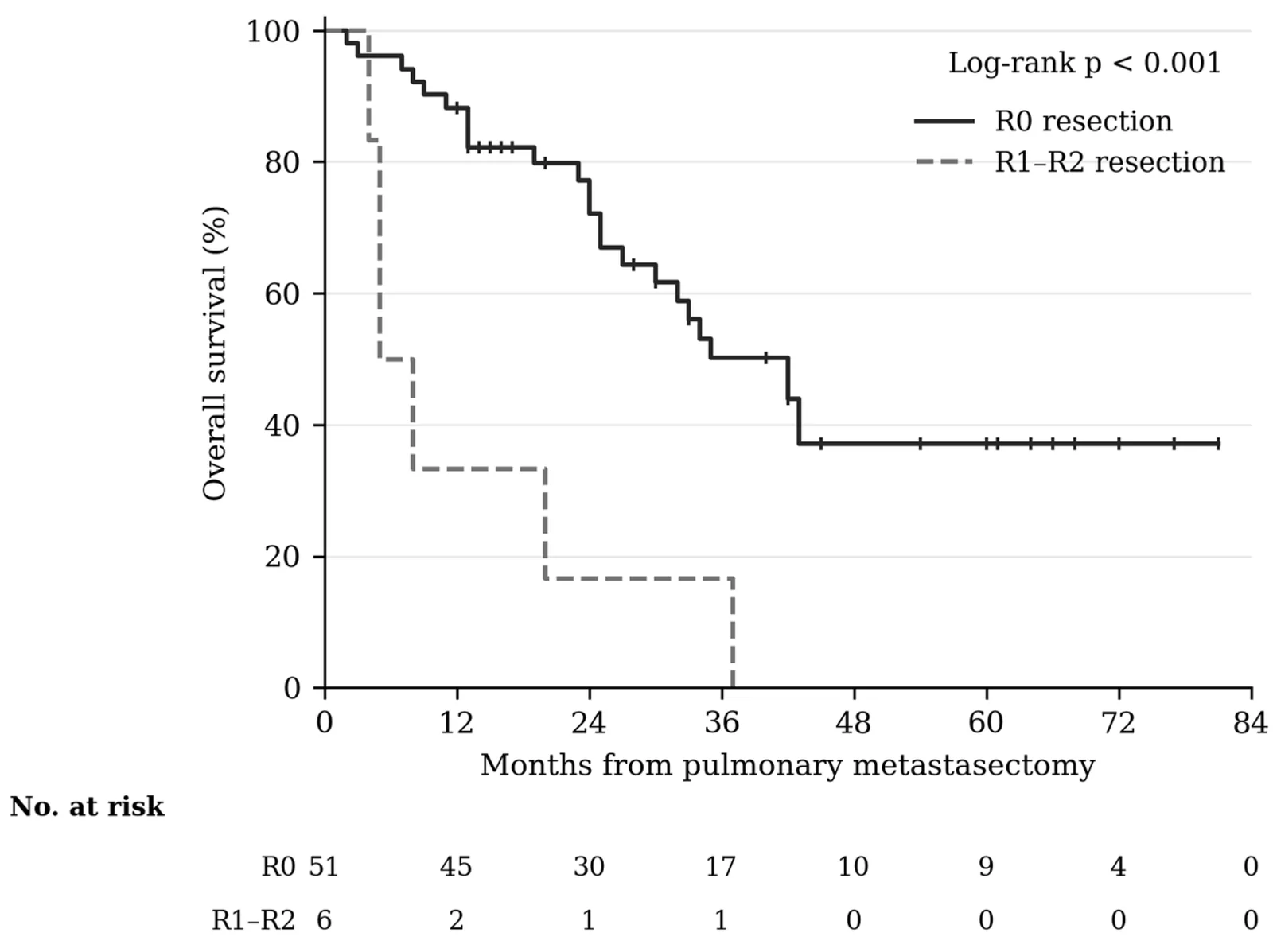
Overall survival according to completeness of resection (R0 vs. R1–R2). Vertical marks denote censored observations.

**Figure 3:**
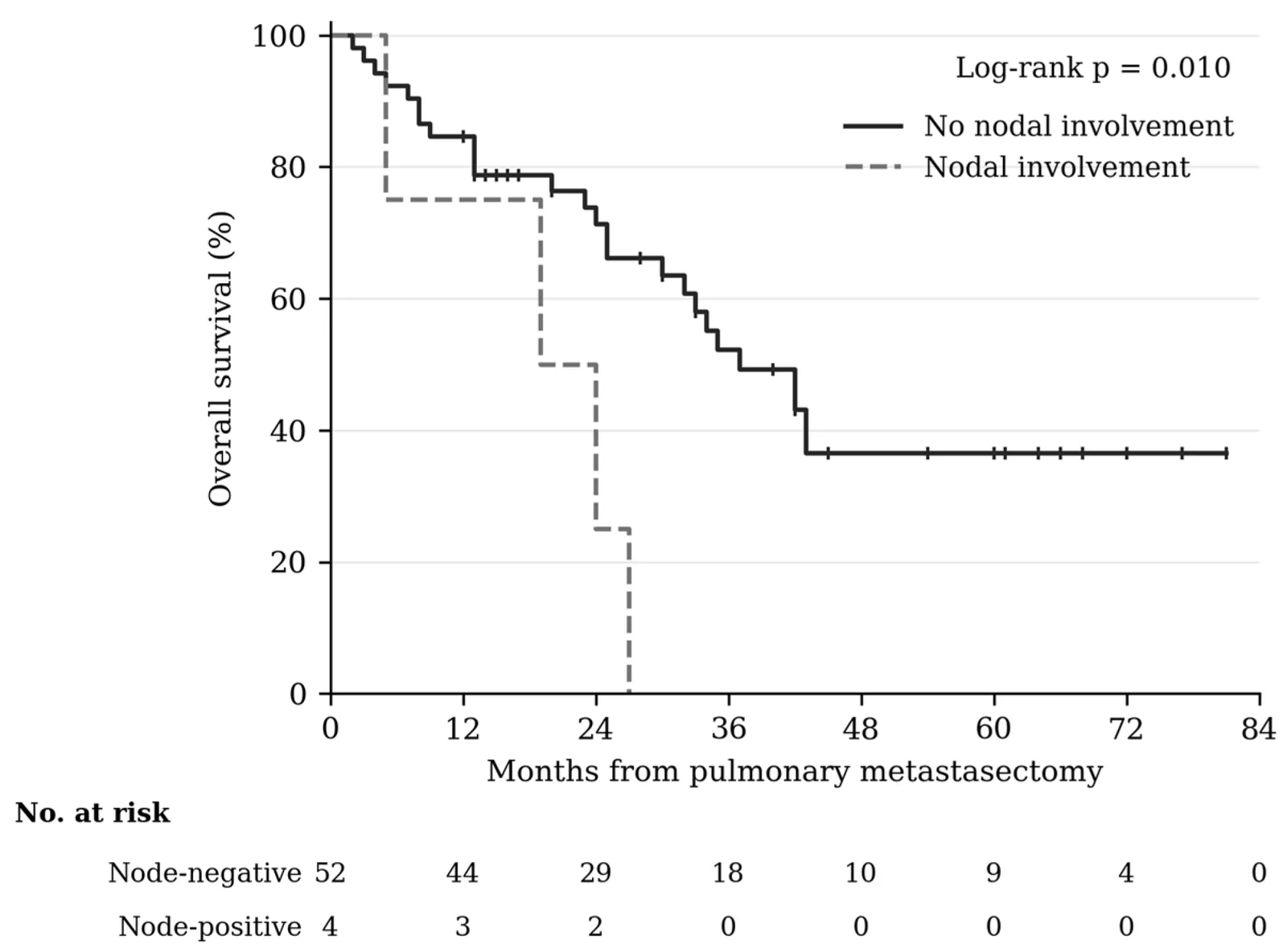
Overall survival according to hilar or mediastinal nodal involvement, among patients in whom nodal involvement was assessed at the index metastasectomy. One patient could not be unambiguously classified for nodal involvement and is not included in this figure (56 of the 57 patients are shown).

The strongest univariable association was completeness of resection (Figure 2): median OS was 42.0 months after R0 resection versus 8.0 months after R1–R2 resection (*p* = 0.0001), with 5-year OS of 37.1% and 0%, respectively. Margin positivity predicted worse OS (median 13.0 vs. 35.0 months; *p* = 0.006), and nodal involvement likewise identified patients with worse OS (median 19.0 vs. 37.0 months; *p* = 0.010), with no node-positive patient surviving to three years (Figure 3).

**Figure 4:**
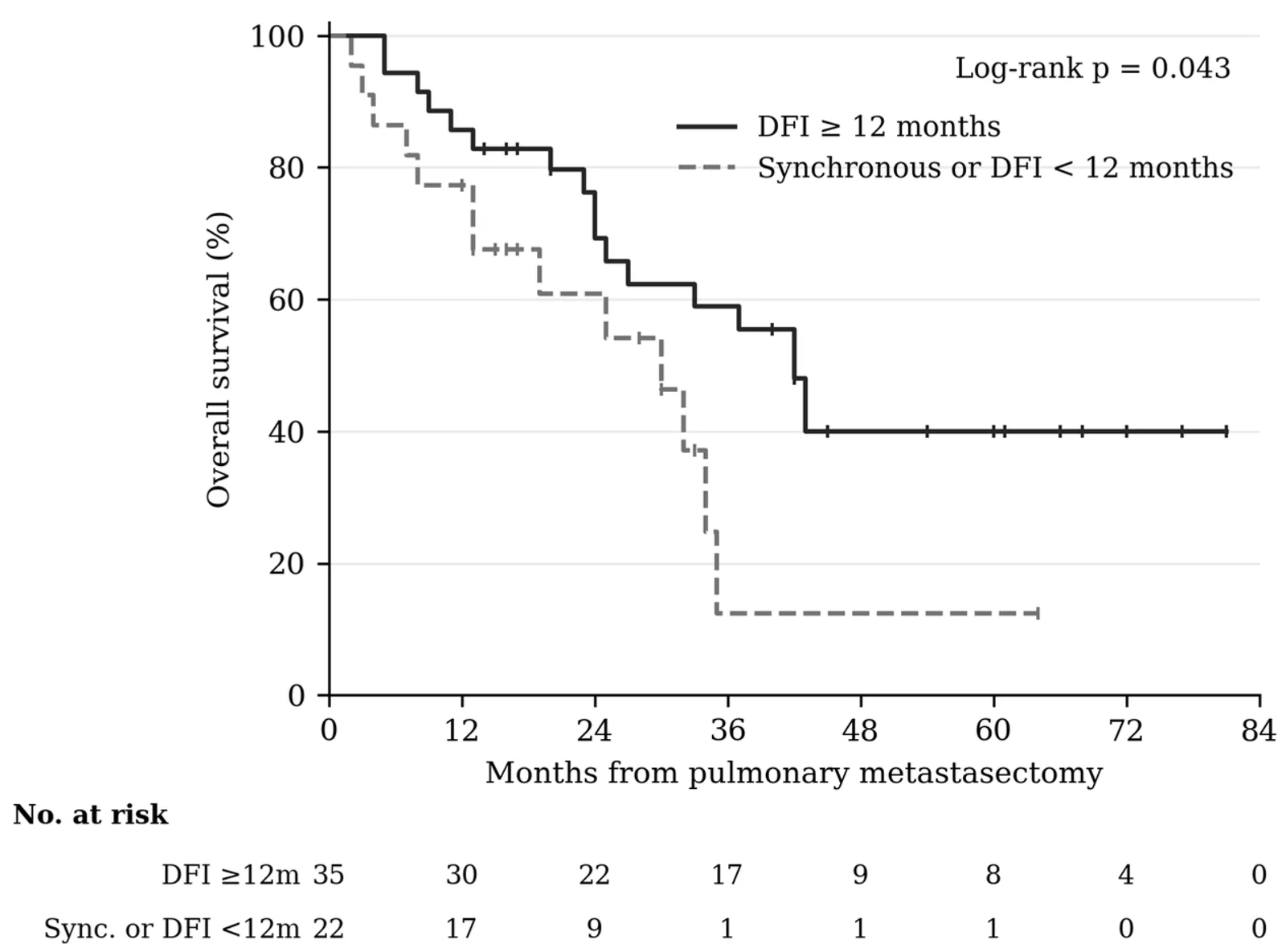
Overall survival according to timing of metastatic appearance, comparing a disease-free interval of at least 12 months with early appearance (synchronous disease or a disease-free interval below 12 months).

Within metachronous disease, a DFI below 12 months predicted shorter OS (median 19.0 vs. 42.0 months; *p* = 0.006; 3-year OS 0% vs. 58.9%). Grouping synchronous disease with a DFI below 12 months as a single category of early metastatic appearance gave a median OS of 30.0 versus 42.0 months (*p* = 0.043) and 3-year OS of 12.4% versus 58.9% (Figure 4). Resection of more than three nodules correlated with shorter median OS (24.0 vs. 37.0 months) and 5-year OS of 0% versus 38.9%, without reaching significance (*p* = 0.13). Primary tumour site (extremity vs. non-extremity) had no effect on OS (median 33.0 vs. 42.0 months; *p* = 0.658). Neither neoadjuvant chemotherapy (32.0 vs. 37.0 months; *p* = 0.595) nor neoadjuvant radiotherapy (32.0 vs. 35.0 months; *p* = 0.799) nor adjuvant radiotherapy (35.0 vs. 34.0 months; *p* = 0.596) was associated with OS; adjuvant chemotherapy showed a non-significant univariable trend towards worse OS (24.0 vs. 42.0 months; *p* = 0.066).

### 3.7. Disease-Free Survival: Univariable Analysis

**Figure 5:**
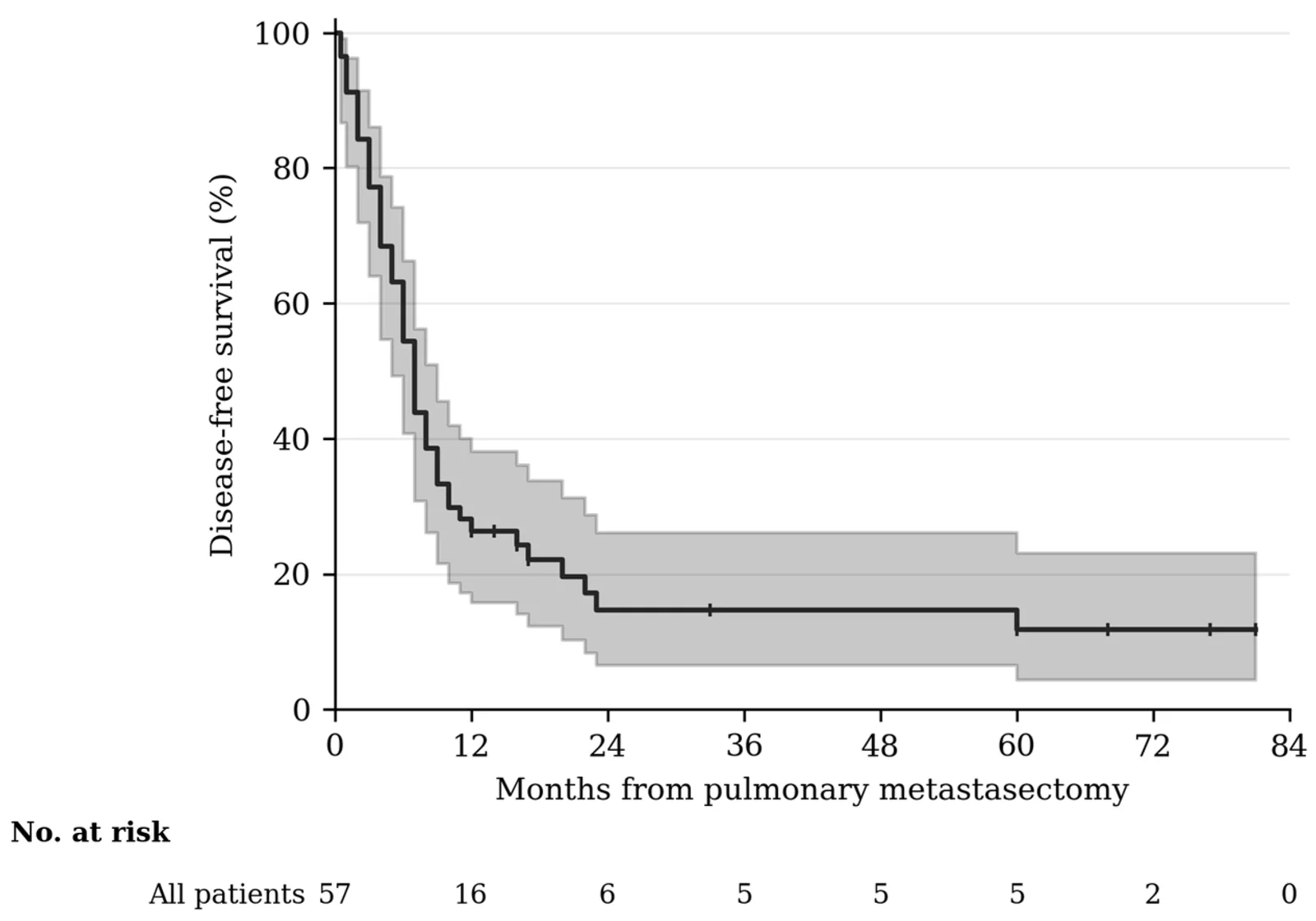
Kaplan–Meier estimate of disease-free survival for the whole cohort (with shadows lines reflecting 95% CI). Vertical marks denote censored observations.

Median DFS for the whole cohort was 7.0 months, with 1- and 3-year DFS of 26.3% and 14.7% (Figure 5). Disease-free survival is reported to three years throughout, in line with the ascertainment period for recurrence.

Histological group had no effect (8.0 vs. 7.0 months for bone vs. soft-tissue sarcoma; *p* = 0.89), nor did surgical approach (*p* = 0.75) or number of nodules resected (*p* = 0.26). Incomplete resection (median 3.0 vs. 7.0 months; *p* < 0.0001) and margin positivity (median 3.0 vs. 7.0 months; *p* = 0.002) were both strongly linked to early recurrence, no patient in either adverse group remaining recurrence-free at one year.

**Figure 6:**
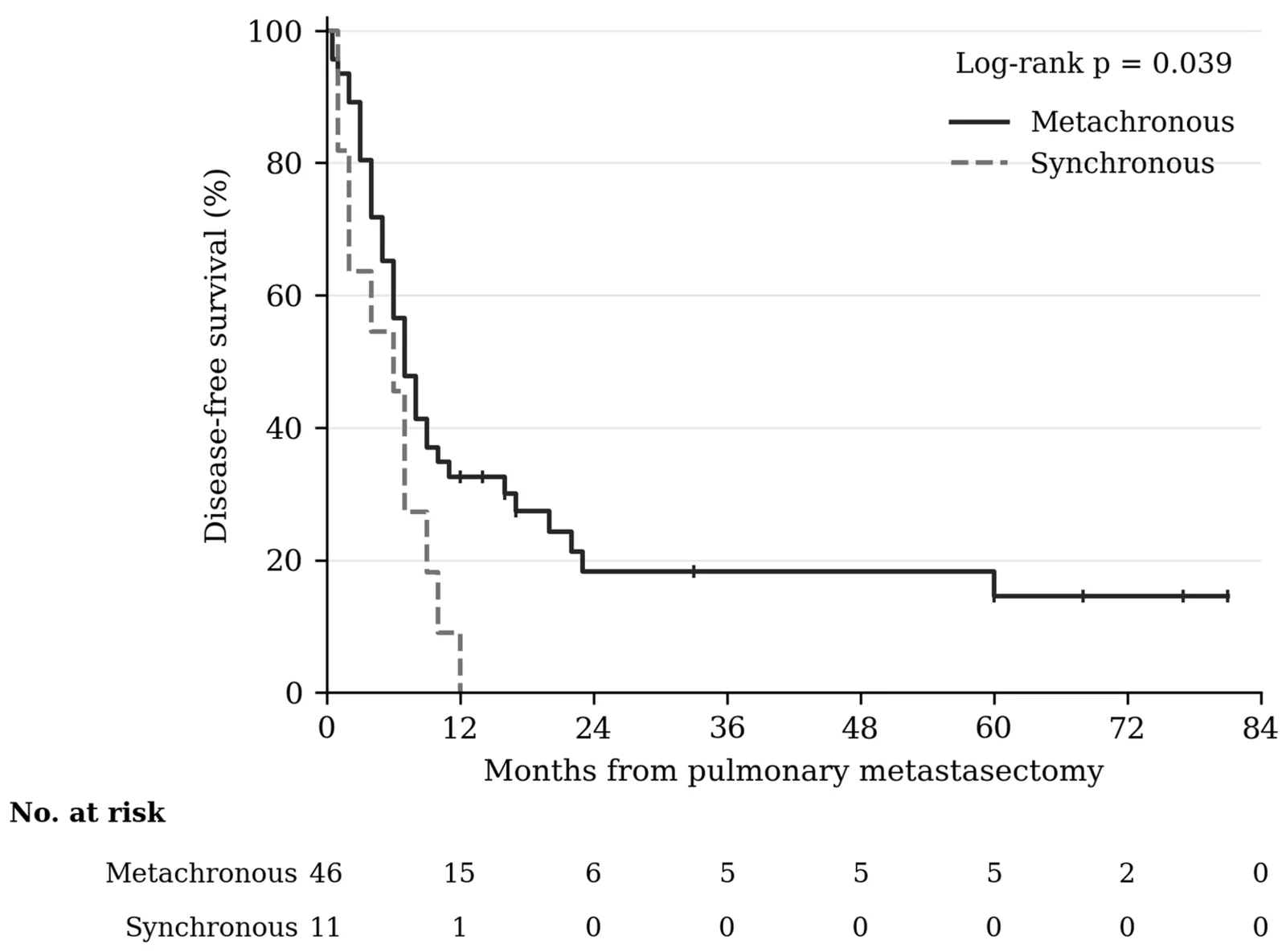
Disease-free survival according to timing of metastases (synchronous vs. metachronous). Vertical marks denote censored observations.

Synchronous presentation correlated with worse DFS (median 6.0 vs. 7.0 months; *p* = 0.039; Figure 6): 1- and 3-year DFS were 32.6% and 18.2% in metachronous disease versus 0% at both time points in synchronous disease. Among metachronous patients, the disease-free interval did not discriminate (median 4.0 vs. 8.0 months; *p* = 0.27), and neither did nodal involvement (*p* = 0.22). Primary tumour site likewise had no effect on DFS (median 5.0 vs. 8.0 months; *p* = 0.256), nor did neoadjuvant radiotherapy (*p* = 0.684) or adjuvant radiotherapy (*p* = 0.844). Adjuvant chemotherapy showed a non-significant univariable trend towards shorter DFS (7.0 vs. 7.0 months; *p* = 0.065); neoadjuvant chemotherapy showed a similar trend that met the pre-specified *p* < 0.2 screening threshold (7.0 vs. 8.0 months; *p* = 0.087) and was therefore carried forward into a post hoc sensitivity analysis (Section 3.8).

### 3.8. Multivariable Analysis

Two variables were retained in the final Cox model for overall survival (Table 1), fitted on all 57 patients with 31 deaths: incomplete resection, the factor most strongly associated with adverse outcome, and early metastatic appearance. The number of nodules resected was screened into the model but did not improve fit by the Akaike criterion and was not retained.

Two variables were likewise retained in the final model for disease-free survival (Table 2), fitted on all 57 patients with 48 recurrence events: incomplete resection, again the factor most strongly associated with outcome, and synchronous presentation. Adding a separate term for the disease-free interval within metachronous disease worsened model fit and was not retained.

**Figure 7:**
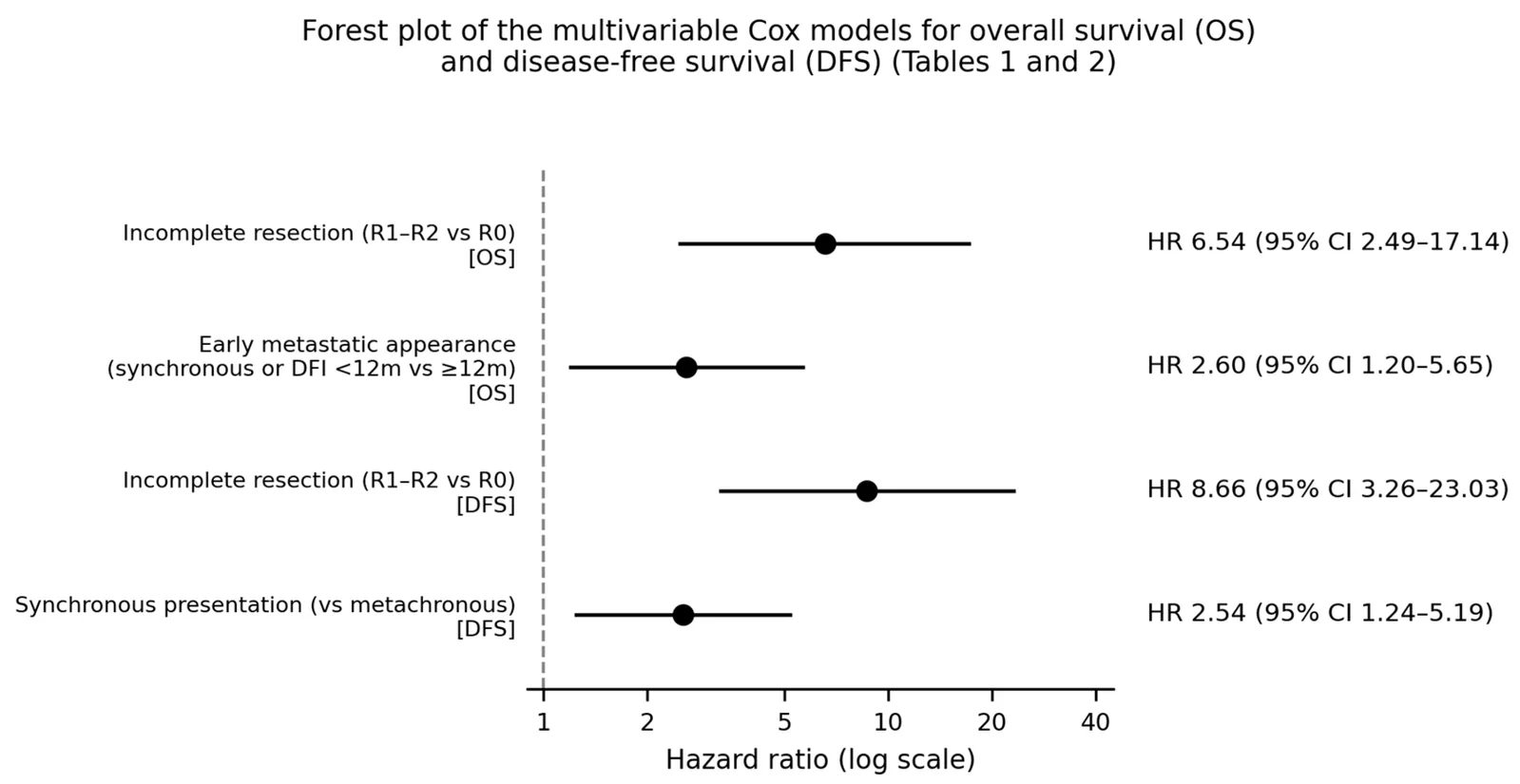
Forest plot of the multivariable Cox models for overall survival (Table 1) and disease-free survival (Table 2). Points show the hazard ratio and lines the 95% confidence interval, on a logarithmic axis; the dashed line marks a hazard ratio of 1. Added in response to peer review.

Sensitivity analysis (post hoc): Neoadjuvant and adjuvant chemotherapy and radiotherapy were not part of the pre-specified candidate list in Section 2.6; we tested them post hoc against the two final models above. Adding adjuvant chemotherapy did not materially improve model fit for either endpoint (ΔAIC < 1 for both OS and DFS) and it was not independently associated with either endpoint (OS: HR 1.81, 95% CI 0.88–3.73, *p* = 0.11; DFS: HR 1.69, 95% CI 0.92–3.09, *p* = 0.09). Adding neoadjuvant chemotherapy to the disease-free survival model improved fit by the Akaike criterion (ΔAIC = 5.5) and neoadjuvant chemotherapy was independently associated with shorter disease-free survival (HR 2.48, 95% CI 1.31–4.68, *p* = 0.005); in this expanded model the hazard ratios for incomplete resection (HR 13.59, 95% CI 4.74–38.99) and synchronous presentation (HR 3.08, 95% CI 1.48–6.42) increased further. This increase should be interpreted cautiously: with only 48 recurrence events, the expanded three-variable model has limited precision, and the marked rise in the incomplete-resection estimate may partly reflect model instability rather than a true change in the underlying effect. This sensitivity model is reported for transparency and is discussed in Section 4; it does not replace the pre-specified two-variable model of Table 2, because neoadjuvant chemotherapy was not part of the original analysis plan and its selection here is exploratory rather than confirmatory (Figure 7).

Nodal involvement, excluded from both multivariable models for the reasons stated in Section 2.6, showed a univariable association with survival (*p* = 0.010), with no node-positive patient surviving to three years.

The proportional hazards assumption was satisfied in both final models, and no covariate showed evidence of time-varying effect.

Histological subtype, surgical approach and type of resection were not independently associated with either endpoint. Adjuvant chemotherapy and radiotherapy showed no independent association with OS or DFS on univariable or sensitivity analysis; neoadjuvant chemotherapy, although not part of the pre-specified candidate list, was independently associated with shorter disease-free survival in the post hoc sensitivity model described above (Section 4).

### 3.9. Repeat Metastasectomy

Eleven repeat metastasectomies were performed in nine patients (four men, five women; mean age 55.8 ± 7.6 years). No repeat procedure was indicated because of an R1–R2 resection at the previous operation; in every case, the indication was pulmonary recurrence of the disease. Seven patients had a soft-tissue sarcoma and two a bone sarcoma, and all had unilateral disease; five procedures were redo-VATS and five rethoracotomies, with one was a thoracotomy after a previous VATS. Lymphadenectomy was performed in the four patients who had not undergone it previously, and no nodal station was involved. R0 resection was achieved in 10 of 11 procedures (91%), mean hospital stay was 4.3 ± 2.4 days, and two procedures (18.1%) were followed by a complication (one haemothorax, one fever resolving with antibiotics), with no readmission or 30-day mortality. Recurrence followed seven procedures; four patients remained recurrence-free and four died (44.4%). Given the size of this subgroup, it is described without formal survival estimation.

## 4. Discussion

In this consecutive cohort of 57 patients undergoing 80 pulmonary metastasectomies for sarcoma, two factors were independently related to each endpoint: completeness of resection and early metastatic appearance for overall survival, and completeness of resection and synchronous presentation for disease-free survival. The number of metastases resected, histological subtype, surgical approach and extent of parenchymal resection showed no independent effect on either endpoint. Perioperative systemic therapy showed no independent association on univariable or post hoc sensitivity analysis, with the exception of neoadjuvant chemotherapy, which was independently associated with shorter disease-free survival in a post hoc sensitivity analysis discussed below. Occult hilar or mediastinal nodal involvement predicted worse survival on univariable analysis but was deliberately kept out of the multivariable models, for the reasons set out below. The perioperative profile was favourable—13.8% morbidity, no 30-day readmission and no 30-day mortality—confirming that the procedure is safe when indicated within a multidisciplinary framework, in line with other contemporary sarcoma series [11].

Completeness of resection. The strength of the association between R0 resection and survival in our model is consistent with essentially the entire published experience: the International Registry of Lung Metastases reported a median survival of 14 months after incomplete resection, worse than that of patients combining the two other classical adverse factors [2], and comparable gradients have been described in soft-tissue sarcoma by Billingsley et al. (33.5 vs. 16.4 months) [12] and by Smith et al. (22, 11.5 and 9.5 months for R0, R1 and R2) [13], with margin involvement the strongest negative predictor in the Australian series of Dear et al. [14]. Not all series agree: Lin et al. concluded that margin negativity beyond macroscopic clearance did not affect prognosis [15]. The magnitude of the effect we observed is large for both endpoints and the confidence intervals correspondingly wide, reflecting the small number of non-radical procedures (n = 7), but the direction is unambiguous. Because completeness of resection is not randomly assigned, the possibility that patients in whom resection proved incomplete already had more extensive or biologically more aggressive disease—confounding by indication—cannot be excluded by this design. In a post hoc comparison, the largest resected nodule was ≥3 cm more often in patients with an R1–R2 result than in those achieving R0 (4/6, 66.7% vs. 13/51, 25.5%; Fisher’s exact *p* = 0.058), whereas bilateral disease, number of nodules resected, synchronous presentation and primary tumour size did not differ between the two groups. This comparison is limited by the small number of patients with an incomplete resection (n = 6) and by the fact that only a limited set of potential confounders could be assessed from the available data; in particular, histological grade—a recognised determinant of metastatic behaviour—was not systematically recorded (Limitations) and could not be examined, so residual or unmeasured confounding cannot be excluded. This pattern is compatible with both explanations—incomplete resection may itself worsen outcome, and/or patients with larger, less favourably situated disease may be both harder to resect completely and biologically more aggressive—and our design cannot separate them; the term ‘modifiable’ used elsewhere in this paper should be read as describing a technical outcome the surgical team can influence through case selection, staging and intraoperative judgement, not as a guarantee that achieving R0 in every patient would reproduce the survival difference observed here. The practical implication is that intraoperative assessment of margins deserves more systematic attention in metastasectomy than it currently receives, particularly given the growth patterns described by Welter et al., in which sarcoma metastases showed the highest incidence of pleural infiltration (31.2%) of any histology [16]; where a parenchyma-sparing resection is chosen, a margin of 5–10 mm is generally recommended [17].

Nodal involvement. Occult nodal involvement was found in 6.5% of assessed procedures and no node-positive patient survived to three years (median 19.0 vs. 37.0 months; *p* = 0.010 univariably). None of these cases had been suspected on the preoperative work-up, which comprised thin-section thoracic CT in every patient and EBUS or PET/CT where imaging raised the suspicion of nodal disease. We deliberately refrained from carrying this variable into the multivariable models: with four events, any adjusted hazard ratio would be subject to sparse-data bias and reported with a confidence interval too wide to inform practice. The univariable finding is nonetheless clinically meaningful and supports considering hilar–mediastinal lymphadenectomy at metastasectomy where feasible, primarily for staging rather than as an established therapeutic intervention; we present this as a hypothesis-generating observation drawn from four events rather than as a firm treatment recommendation, and it requires confirmation in larger series before it should influence practice more broadly. The argument is one of staging rather than of therapeutic benefit: nodal involvement is not obtainable preoperatively in this setting, cannot be inferred from imaging, and identifies a subgroup whose prognosis is sufficiently different to alter subsequent systemic management and surveillance intensity. This position is consistent with Kamiyoshihara et al. [18] and the review by Reinersman and Wigle [19], and with the observation that nodal dissection during metastasectomy remains inconsistently performed across European practice [20].

Disease-free interval and synchronicity. Timing of metastatic appearance was retained in both multivariable models, though not in the same parameterisation. Because synchronous disease is by definition the extreme of a short disease-free interval, synchronicity and dichotomised DFI were never entered as separate covariates in the same model, which would have introduced structural collinearity and invited the misreading that two separable effects had been demonstrated. For overall survival, grouping synchronous disease with a DFI below 12 months into a single category of early metastatic appearance produced the best-fitting model. For disease-free survival, synchronicity alone was retained: among metachronous patients the disease-free interval did not discriminate (median DFS 4.0 vs. 8.0 months; *p* = 0.27), whereas no patient with synchronous disease was recurrence-free at one year against 32.6% of metachronous patients.

Published series are not consistent on this point, and the disagreement is worth stating explicitly. Most report both shorter disease-free and shorter overall survival in patients with synchronous metastases, and Buddingh et al., in pulmonary metastasised high-grade osteosarcoma, likewise linked a longer interval to metastatic appearance with better overall survival [21]; others find no difference in overall survival between synchronous and metachronous presentation at all [22]. Nor is there consensus on the disease-free interval threshold: 25 months has been proposed in soft-tissue sarcoma [13], 12 months for pulmonary and 24 months for extrapulmonary metastases [7], and 16 months in a Brazilian series [23], while Briccoli et al. and Kim et al. both found 12 months discriminant [8,9]. Against results this heterogeneous, the defensible reading of our data is not that they settle the threshold, but that in a long consecutive series accrued under a single, unchanged indication policy, early appearance of pulmonary disease consistently identified a poor-prognosis group. Knowing what one’s own series shows is, in this setting, more useful than importing a cut-off derived from a cohort managed differently. Both statements rest on eleven synchronous patients and require confirmation in larger cohorts.

Number of metastases. Resection of more than three nodules was followed by a shorter median survival (24.0 vs. 37.0 months) and by 5-year survival of 0% against 38.9%, but the difference did not reach significance (*p* = 0.13) and the variable did not improve the fit of the multivariable model. A cut-off of three lesions recurs across retrospective series, though its statistical robustness has repeatedly been questioned. Our data are compatible with a real effect that this cohort, with eleven patients above the threshold, is underpowered to demonstrate; they do not support using nodule count alone as a contraindication to surgery.

Perioperative systemic therapy. Neither neoadjuvant nor adjuvant radiotherapy, nor adjuvant chemotherapy, showed a univariable or independent association with OS or DFS (Section 3.6, Section 3.7 and Section 3.8). Neoadjuvant chemotherapy did not reach significance in univariable analysis for either endpoint, but its univariable *p*-value for DFS (0.087) met the *p* < 0.2 threshold we pre-specified for the rest of the model, and in a post hoc sensitivity analysis it was independently associated with shorter DFS (HR 2.48, 95% CI 1.31–4.68). We interpret this cautiously rather than as evidence that neoadjuvant chemotherapy causes early recurrence: patients selected for neoadjuvant treatment of the primary tumour are, by the indication for that treatment, more likely to have had larger, higher-grade or otherwise more aggressive primary disease, and confounding by indication is at least as plausible an explanation as a direct treatment effect. We did not have histological grade recorded systematically enough to adjust for it directly (Limitations), which limits our ability to disentangle the two explanations further. The concurrent rise in the hazard ratio for incomplete resection in this expanded model (from 6.54 to 13.59) should be interpreted with similar caution, as it may reflect instability in a model with a limited number of events rather than a genuine change in effect. We report this association for transparency because it emerged once an omission identified during peer review was corrected, but we consider it hypothesis-generating rather than confirmatory.

Histological subtype. We found no difference in OS or DFS between bone and soft-tissue sarcoma, or between soft-tissue subtypes. The literature is divided: Gusho et al., using SEER data with propensity matching, found a longer disease-free interval in bone sarcoma [24] and Lin et al. reported longer survival for osteosarcoma and leiomyosarcoma than for liposarcoma or synovial sarcoma [15], while other series find no such differences [25,26]. With 11 bone sarcomas and more than 15 soft-tissue subtypes represented, our cohort has no power to resolve this question, and the absence of a difference should be read as uninformative rather than as evidence against one.

Surgical approach. Neither OS nor DFS differed between thoracotomy and VATS, in a cohort where the proportion of minimally invasive procedures rose from 0% to 46% between the two study periods, mirroring the trajectory reported elsewhere in Europe and in the European Society of Thoracic Surgeons database [27,28,29]. The classical objection to VATS is the radiological–pathological discrepancy, which is greater in sarcoma than in any other primary: Kang et al. reported a per-nodule sensitivity of multidetector CT of 97% in non-osteosarcoma patients but only 34% in sarcoma, with a negative predictive value of 38% [30]. Earlier work concluded on this basis that complete resection required an open approach, and prospective sequential studies confirmed that palpation finds additional nodules missed at thoracoscopy [31,32,33,34]. None of these studies, however, demonstrated that the additional nodules translate into a survival difference, which is the question that matters clinically and the one our data address, albeit with limited power and without randomisation; series comparing approaches directly have found no survival penalty for VATS [35]. Our position is that the choice of approach should be individualised, with open exploration retained where imaging burden and histology suggest a high probability of occult disease, and that this remains an area where prospective comparative data are genuinely needed [36].

Repeat metastasectomy. Eleven redo procedures in nine patients achieved R0 resection in 91%, with 18% morbidity and no mortality, and four of the nine remained recurrence-free. These numbers are too small for inference, but they are consistent with a substantial body of evidence that repeat metastasectomy is feasible and safe in selected patients and can be followed by prolonged survival when disease remains confined to the lung [37,38,39,40]. Chudgar et al., in the largest series available, reported longer survival among patients selected for repeat resection than among those managed non-operatively after recurrence, while cautioning that selection accounts for much of that difference [37]; Hamaji et al. reached a comparable conclusion in a mixed bone and soft-tissue cohort [38], and similar findings have been reported in soft-tissue sarcoma [39] and in osteosarcoma of the extremities [40]. As at first metastasectomy, completeness of resection again showed the strongest association with outcome, which supports applying the same selection criteria to the second operation as to the first.

Model performance. Discrimination of the final Cox models was modest: concordance was 0.67 for the overall-survival model and 0.64 for the disease-free-survival model, barely better than chance. This is unsurprising given the small number of covariates retained and the exploratory, hypothesis-generating purpose of the variable-selection strategy (Section 2.6), but it means neither model should be used as an individual-level prediction tool in its current form; the practical claims we draw from them—that completeness of resection and timing of metastatic appearance matter for prognosis—should be read as qualitative associations rather than as a validated risk score.

Limitations. The principal limitations are inherent to the design. Selection, information and confounding biases follow from the partly retrospective, uncontrolled nature of the study: patients underwent surgery precisely because they were judged likely to benefit, and no comparator group of medically managed patients is available. For the same reason, this study describes prognosis within an operated population and identifies factors associated with better or worse outcome after surgery; it cannot establish that metastasectomy itself, rather than the underlying disease biology or non-surgical management, is responsible for the outcomes observed, nor can it determine which patients would, or would not, have benefited from an operation they did not have. The sample size, though representative of a reference centre for a rare disease, limits power, and several of the associations we report have wide confidence intervals. Variable selection at *p* below 0.2 followed by Akaike-based model selection retains variables that are not statistically significant, which is defensible for hypothesis generation but means the reported hazard ratios should be treated as estimates requiring external validation rather than as calibrated risk coefficients; we have accordingly restricted the term prognostic factor to associations whose confidence interval excludes unity. The number of non-radical resections (n = 7), of node-positive patients (n = 4) and of synchronous presentations (n = 11) is small, which limits the precision of the corresponding estimates and was the reason for excluding nodal involvement from the multivariable models. Functional and comorbidity data—ASA class, ECOG performance status, body mass index, smoking history, Charlson index and respiratory comorbidity—were not systematically recorded in the study database and could not be entered as covariates; their absence limits any assessment of operative selection and should be addressed in prospective registry work. Histological grade, one of the best-established determinants of metastatic behaviour and post-metastasectomy survival in sarcoma, was likewise not systematically recorded across the study period and could not be analysed; its absence is a limitation shared with much of the pre-existing single-centre literature and should be prioritised in future prospective data collection. Recurrence status was systematically ascertained to three years, so disease-free survival beyond that horizon is not reported. Finally, the histological heterogeneity intrinsic to sarcoma means that the cohort aggregates diseases with different natural histories. The proportion of procedures performed by VATS rose from 0% to 46.3% between the retrospective and prospective periods, a shift that could in principle reflect broader era effects (imaging technology, referral patterns, systemic therapy) rather than the surgical approach alone; overall survival did not differ significantly between the two periods (median 43.0 vs. 27.0 months, retrospective vs. prospective; *p* = 0.152), nor did disease-free survival (9.0 vs. 7.0 months; *p* = 0.351), which does not support a strong era-related improvement coinciding with the adoption of VATS, though a smaller effect cannot be excluded with this sample size.

Perspectives. Resolving these questions requires cohorts larger than any single centre can accrue. National and European registries of sarcoma pulmonary metastasectomy, including the registry currently under development by the Spanish Association of Surgeons, offer the most realistic route to validating the factors identified here and to answering the comparative questions a single-centre series cannot. Current European and Spanish guidance already frames metastasectomy as one component of a multimodal strategy in oligometastatic sarcoma rather than as a stand-alone treatment [5,6,41]. Within this framework, non-surgical local-control alternatives such as thermal ablation have been proposed specifically for patients in whom metastasectomy is not feasible, with survival outcomes reported as broadly comparable in selected series [42]. A recent referral-centre cohort reached convergent conclusions on completeness and type of resection, reinforcing the value of consistent, protocol-driven practice within reference networks [43].

## 5. Conclusions

Complete (R0) resection showed the strongest association with both overall and disease-free survival after pulmonary metastasectomy for sarcoma, and is the only identified factor under the surgeon’s direct control. This should be interpreted as the strongest identified association rather than as proof of a causal, modifiable effect: confounding by indication cannot be excluded, as detailed in Section 4 and Limitations. Early appearance of pulmonary metastases—synchronous disease, or a disease-free interval shorter than twelve months—identifies patients in whom surgery alone is unlikely to control the disease and in whom the indication should be weighed particularly carefully. Occult hilar or mediastinal nodal involvement, which cannot be predicted from preoperative imaging, carried a clear adverse univariable association and supports systematic lymphadenectomy for the prognostic information it yields. Neither histological subtype, surgical approach, extent of parenchymal resection nor the number of nodules resected independently affected outcome, nor did adjuvant chemotherapy or radiotherapy; neoadjuvant chemotherapy was independently associated with shorter disease-free survival in a post hoc sensitivity analysis that requires confirmation (Section 4). Discrimination of both multivariable models was modest (concordance 0.67 for OS and 0.64 for DFS), so these estimates should be read as hypothesis-generating rather than as a validated risk-prediction tool, so patient selection within a multidisciplinary team, rather than any single technical variable, remains the decisive factor. When appropriately indicated, pulmonary metastasectomy, including repeat metastasectomy, was safe in our experience, with low morbidity and no perioperative mortality.

## Figures and Tables

**Table 1:** Multivariable Cox model for overall survival.

*p*	95% CI	Hazard Ratio	Variable
0.0001	2.49–17.14	6.54	Incomplete resection (R1–R2 vs. R0)
0.015	1.20–5.65	2.6	Early metastatic appearance (synchronous or DFI < 12 months, vs. DFI ≥ 12 months)

Model fitted on all 57 patients with 31 deaths; concordance 0.67. Nodal involvement was excluded a priori (Section 2.6). Number of nodules resected was screened in but not retained by the Akaike criterion. Adjuvant chemotherapy was screened in as a post hoc sensitivity analysis (Section 3.8) and was not retained.

**Table 2:** Multivariable Cox model for disease-free survival.

*p*	95% CI	Hazard Ratio	Variable
<0.0001	3.26–23.03	8.66	Incomplete resection (R1–R2 vs. R0)
0.011	1.24–5.19	2.54	Synchronous presentation (vs. metachronous)

Model fitted on all 57 patients with 48 recurrence events; concordance 0.64. A separate term for disease-free interval within metachronous disease did not improve model fit and was not retained. Adjuvant chemotherapy was screened in as a post hoc sensitivity analysis (Section 3.8) and was not retained; neoadjuvant chemotherapy was screened in post hoc and was independently associated with shorter disease-free survival in an expanded, exploratory model reported separately in Section 3.8.

**Table 3:** Patient and primary tumour characteristics (n = 57 patients).

n (%) or Mean ± SD	Variable
34 (59.6)	Male sex
23 (40.4)	Female sex
52.8 ± 14.0	Age at metastasectomy, years
46 (80.7)	Soft-tissue sarcoma
11 (19.3)	Bone sarcoma
16 (35)	Leiomyosarcoma (of soft-tissue sarcomas)
5 (11)	Synovial sarcoma (of soft-tissue sarcomas)
4 (9)	Pleomorphic sarcoma (of soft-tissue sarcomas)
4 (9)	Epithelioid sarcoma (of soft-tissue sarcomas)
7 (63.6)	Primary in extremity—bone sarcoma
24 (52.2)	Primary in extremity—soft-tissue sarcoma
10.3 ± 6.3	Primary tumour size, cm (n = 54)
20 (35.1)	Neoadjuvant chemotherapy
9 (15.8)	Neoadjuvant radiotherapy
25 (43.9)	Adjuvant chemotherapy
28 (49.1)	Adjuvant radiotherapy
46 (80.7)	Metachronous metastases
11 (19.3)	Synchronous metastases
35 (76.1)	Disease-free interval ≥ 12 months (of metachronous)
42 (73.7)	Unilateral disease
15 (26.3)	Bilateral disease

**Table 4:** Characteristics of the 80 metastasectomy procedures.

n (%)	Variable
50 (62.5)	Thoracotomy
20 (25.0)	VATS
5 (6.25)	Rethoracotomy
5 (6.25)	Redo-VATS
44 (55.0)	One nodule resected
19 (23.8)	Two to three nodules resected
13 (16.2)	Four to ten nodules resected
4 (5.0)	More than ten nodules resected
55 (68.8)	Largest nodule < 3 cm
25 (31.2)	Largest nodule ≥ 3 cm
73 (91.2)	R0 resection
5 (6.2)	R1 resection
2 (2.5)	R2 resection
59 (73.8)	Hilar–mediastinal lymphadenectomy
2 (2.5)	Nodal sampling
19 (23.8)	No nodal assessment
4 (6.5)	Nodal involvement (of 61 assessed)
11 (13.8)	Any complication
0 (0)	30-day readmission
0 (0)	30-day mortality

## Data Availability

The data presented in this study are available on request from the corresponding author. The data are not publicly available owing to privacy and ethical restrictions.

## Supplementary Materials

The following supporting information can be downloaded at: https://www.mdpi.com/article/10.3390/cancers18183054/s1, Table S1: STROBE checklist for cohort studies.

## Abbreviations

CI	Confidence interval
DFI	Disease-free interval
DFS	Disease-free survival
DLCO	Diffusing capacity of the lung for carbon monoxide
FEV1	Forced expiratory volume in one second
HR	Hazard ratio
OS	Overall survival
VATS	Video-assisted thoracoscopic surgery
