# Supplementary material for: Clinicopathological Prognostic Factors After Pulmonary Metastasectomy for Bone and Soft-Tissue Sarcoma: A Single-Centre Retrospective–Prospective Cohort Study
Source: Cancers (Basel). 2026 Sep 20;18(18):3054. doi: 10.3390/cancers18183054 (PMC13604907; doi:10.3390/cancers18183054)
Supplement: Supplementary file 1 [file cancers-18-03054-s001.zip › cancers-4566961-supplementary.pdf]

Table S1. STROBE checklist for cohort studies

Manuscript: Clinicopathological Prognostic Factors After Pulmonary Metastasectomy for Bone and Soft-Tissue Sarcoma: A Single-Centre Retrospective–Prospective Cohort Study.

Page numbers refer to the final manuscript file submitted with this supplement.

Item No.	Recommendation	Location in manuscript
**Title and abstract**		
1a	State the study design in the title or the abstract.	Title; Abstract, Methods — p. 1
1b	Give a balanced and informative summary of what was done and what was found.	Simple Summary; Abstract — p. 1
**Introduction**		
2	Explain the scientific background and the rationale for the investigation.	Introduction, paras 1–3 — pp. 1–2
3	State the specific objectives, including any prespecified hypotheses.	Introduction, final para — p. 2
**Methods**		
4	Present the key elements of the study design early in the paper.	§2.1 Study Design and Setting — p. 2
5	Describe the setting, locations and relevant dates, including periods of recruitment, exposure, follow-up and data collection.	§2.1 — p. 2
6a	Give the eligibility criteria, and the sources and methods of selection of participants; describe methods of follow-up.	§2.2 Eligibility Criteria; §2.3 Criteria for Pulmonary Metastasectomy — p. 3; follow-up schedule §2.4 — pp. 3–4
6b	For matched studies, give matching criteria and number of exposed and unexposed.	Not applicable — unmatched single-arm cohort
7	Define all outcomes, exposures, predictors, potential confounders and effect modifiers; give diagnostic criteria if applicable.	§2.4 Variables and Definitions — pp. 3–4
8	For each variable of interest, give the sources of data and the details of methods of assessment; describe comparability of assessment methods where there is more than one group.	§2.4 — pp. 3–4; §2.5 Data Sources and Management — p. 4

Item No.	Recommendation	Location in manuscript
9	Describe any efforts to address potential sources of bias.	§2.6 Statistical Analysis (a priori model decisions) — p. 4; Discussion, Limitations — p. 10
10	Explain how the study size was arrived at.	§2.1 — p. 2 (no formal sample size calculation; all consecutive eligible patients)
11	Explain how quantitative variables were handled in the analyses; if applicable, describe which groupings were chosen and why.	§2.4 (dichotomisation of DFI, nodule count, nodule size, FEV1, DLCO) — pp. 3–4
12a	Describe all statistical methods, including those used to control for confounding.	§2.6 — pp. 4–5
12b	Describe any methods used to examine subgroups and interactions.	§2.6 — p. 4; §§3.6–3.7 — p. 6
12c	Explain how missing data were addressed.	§2.5 — p. 4; §3.1 (primary tumour size available for 54 of 57 patients) — p. 5
12d	For cohort studies, explain how loss to follow-up was addressed.	§2.5 — p. 4 (no patient lost to oncological follow-up)
12e	Describe any sensitivity analyses.	§2.6 — pp. 4–5 (alternative parameterisations of metastatic timing compared by the Akaike criterion; nodule count screened and rejected)
**Results**		
13a	Report the numbers of individuals at each stage of the study.	§3.1 — p. 5
13b	Give reasons for non-participation at each stage.	§2.2 (exclusions on pathological review) — p. 3
13c	Consider use of a flow diagram.	Not included — single consecutive series with no staged recruitment
14a	Give characteristics of study participants and information on exposures and potential confounders.	§§3.1–3.3 — pp. 5–6; Table 1 — p. 10; Table 2 — p. 11

Item No.	Recommendation	Location in manuscript
14b	Indicate the number of participants with missing data for each variable of interest.	§3.1 — p. 5
14c	For cohort studies, summarise follow-up time.	§3.6 — p. 6 (median follow-up 54.0 months by reverse Kaplan–Meier)
15	For cohort studies, report numbers of outcome events or summary measures over time.	§3.5 Recurrence; §§3.6–3.7 — p. 6
16a	Give unadjusted estimates and, if applicable, confounder-adjusted estimates and their precision; state which confounders were adjusted for and why.	Univariable: §§3.6–3.7 — p. 6. Multivariable: §3.8 — p. 7; Tables 3 and 4 — pp. 11-12
16b	Report category boundaries when continuous variables were categorised.	§2.4 — pp. 3–4
16c	If relevant, consider translating estimates of relative risk into absolute risk for a meaningful time period.	Not applicable — absolute survival reported directly as Kaplan–Meier estimates, §§3.6–3.7 — p. 6
17	Report other analyses done (subgroups, interactions, sensitivity analyses).	§3.9 Repeat Metastasectomy — p. 7
**Discussion**		
18	Summarise key results with reference to study objectives.	Discussion, opening para — p. 7
19	Discuss limitations, taking into account sources of potential bias or imprecision, and both direction and magnitude of any potential bias.	Discussion, Limitations — p. 9
20	Give a cautious overall interpretation considering objectives, limitations, multiplicity of analyses, results from similar studies and other relevant evidence.	Discussion, thematic blocks — pp. 7–9; Conclusions — p. 9
21	Discuss the generalisability (external validity) of the study results.	Discussion, Limitations and Perspectives — p. 9
**Other information**		
22	Give the source of funding and the role of the funders for the present study and, if applicable, for the original study on which the article is based.	Funding statement — p. 12

Note: item recommendations are given in condensed form. The full official wording of the STROBE Statement checklist is available at www.strobe-statement.org.
